# On the Study of Botulinum Toxin A Actions: Beyond the Neuromodulation Induced by SNAP-25 Cleavage

**DOI:** 10.3390/toxins18100427

**Published:** 2026-10-01

**Authors:** Christine Rasetti-Escargueil, Stefano Palea

**Affiliations:** Humana Biosciences SAS, Prologue Biotech, 516, rue Pierre et Marie Curie, 31670 Labège, France; stefano.palea@humana-biosciences.com

**Keywords:** botulinum neurotoxins, molecular pathways, SNARE cleavage, therapy

## Abstract

The present review is focused on a range of BoNT/A molecular targets and the subsequent modulation of cellular signaling pathways focused on gene regulation, skin and nerve regeneration, vascular effects, and contributions to pain relief. This brings fresh insights to the diversity of BoNT/A mechanisms of action, opening new avenues of research into therapeutic options for patients. A summary of the mechanisms of action of BoNT/A formulations will be presented in parallel with their specific indications, showing the versatility of the BoNTs’ efficiency through different pathways well beyond the canonical SNARE cleavage mechanism. The current research progress is summarized, ranging from the molecular mechanism of BoNT/A involved in the treatment of inflammation, pathological scars, pain, and a range of neurological and vascular disorders via clinical investigations and experimental studies.

## 1. Introduction

BoNTs (botulinum neurotoxins) constitute a family of neurotoxic proteins that are produced by spore-forming, gram-positive, anaerobic bacteria found ubiquitously in the environment and in plants, namely *Clostridium botulinum*, as well as other *Clostridium* spp., such as *Clostridium butyricum*, *Clostridium argentinensis*, *Clostridium Sporogenes*, and *Clostridium baratii* [1,2].

Botulinum toxins are modular proteins that act through three distinct functional modules: a binding domain that attaches to nerve terminals at the neuromuscular junction, a translocation domain mediating delivery of the toxin into the neuronal cytosol, and the enzymatic domain blocking the release of neurotransmitters, producing flaccid paralysis. Botulinum neurotoxins are included in the ”Dirty Dozen” list of potential bioweapon agents compiled by the CDC. This classification underscores their high toxicity and potential for misuse [3].

BoNTs undergo a precise activation pathway where the large 150 kDa single-chain precursor is cleaved by post-translational proteolytic activation through environmental or host proteases into two smaller, functionally distinct chains: a light chain (LC) of 50 kDa and a heavy chain (HC) of 100 kDa. These chains remain connected by a disulfide bond, ultimately creating the potent neurotoxic protein [4,5]. It was recently discovered that the protein α-clostripain was responsible for the cleavage of BoNT/A, leading to a 77-fold increase in toxicity, while the accessory protein NTNH was essential during activation to prevent non-specific degradation of the toxin [6].

BoNTs penetrate synaptic terminals to cleave one of the three SNARE proteins: vesicle-associated membrane protein (VAMP), synaptosomal-associated protein 25 (SNAP-25), or syntaxin. This serotype-dependent action results in the inhibition of neurotransmitter release from synaptic vesicles [7]. The SNARE proteins are critical components of the vesicle docking/fusion process triggering neurotransmitter release; by disrupting this process, the BoNTs cause a progressive descending flaccid paralysis, potentially leading to respiratory failure and death. Apart from this usual mechanism of disrupting neurotransmitter release, the intracellular targets, receptors, and pharmacodynamic/kinetic properties greatly differ among the various BoNT toxinotypes. In addition, the cleavage of SNAP-25 by the light chain of BoNT/A is not exclusively related to binding on specific sites of the LC, but is also the consequence of a dileucine-mediated LCA-septin co-clustering crucial for the stabilization of LCA and its subsequent long-lasting neuroparalytic activity [8]. As opposed to BoNT/A or /B, botulinum neurotoxin type E (BoNT/E) is initially produced as a single-chain toxin. It is subsequently cleaved by host proteases into a di-chain form, which is approximately 100 times more toxic than the single-chain version [9].

In nature, holotoxins are first formed as non-covalently bound progenitor toxin complexes (PTCs). These complexes include non-toxic neurotoxin-associated proteins (NAPs), such as hemagglutinins HA-17, HA-33, and HA-70, along with a non-toxic non-hemagglutinin (NTNHA) protein [10]. All BoNTs are naturally co-expressed with those protective partner proteins (NTNH), forming a 300 kDa complex able to resist acidic and proteolytic attack from the digestive tract, ensuring stabilization and absorption of the BoNTs throughout the intoxication process [11].

The HC domain is subdivided into two functional areas: the translocation domain (HN) and the receptor-binding domain (HC), each weighing 50 kDa. The HC domain has an exceptionally high affinity for binding to presynaptic terminals at the neuromuscular junction and enters the cell through receptor-mediated endocytosis [12]. The translocation domain (HN) is involved in the translocation of the LC into the intracellular compartment, while the acidification within the endosome induces the conformational change within the HN [13]. The entry of the LC into neuronal cells via the vesicle membranes is facilitated by the translocation domain, while the HCn subdomain is responsible for toxin binding through its interaction with lipid microdomains [14,15,16]. Several outstanding questions remain regarding the L-chain translocation across the synaptic vesicle membrane and subsequent molecular events following toxin entry into the cytosol [5]. In addition, a translocation-competent state of the toxin is required for successful LC transport into the neuronal cytosol under acidic conditions [17].

The initial BoNT/A formulation received its license for human therapeutic application in 1984, following Dr. Alan Scott’s discovery of its effectiveness in treating strabismus. A few years later, BoNTs were regulatory approved for the treatment of glabellar lines in the Botox formulation. Botulinum toxins (BoNTs) have become a standard treatment for managing movement disorders, including conditions such as dystonia or spasticity, in individuals affected by bruxism, tremors, tics, myoclonus, restless legs syndrome, or tardive dyskinesia. Moreover, BoNTs have demonstrated effectiveness in addressing common symptoms associated with Parkinson’s disease, such as foot dystonia, freezing of gait, rigidity, or tremor [18]. In addition, due to their high potency, long-term action, and satisfactory safety profile, BoNTs are used [19] empirically in a wide range of ophthalmological, gastrointestinal, urological, orthopedic, dermatological, secretory, and pain disorders.

However, available BoNT therapies are mainly focused to neuronal territories with periodic injections, often causing immunoresistance. Engineering of novel BoNTs will support the development of non-neuronal therapies and facilitate the reduction of immunoresistance. As a result, many studies are still needed to address the underlying mechanisms leading to the very wide therapeutic indications of BoNTs from a mechanistic and therapeutic perspective [19].

A wide range of in vitro and in vivo studies already show that BoNT/A actions are related to cell cycle, apoptosis, neuritogenesis, and gene expression. The present review is focused on various BoNT/A molecular targets and the subsequent modulation of cellular signaling of BoNT/A involved in the treatment of pathological scars, pain, and a range of neurological and vascular disorders. Clinical investigations and experimental studies show the versatility of the BoNTs through myriads of interconnected pathways well beyond the unique SNARE cleavage.

## 2. Beyond SNAP-25 Cleavage

The actions of BoNT/A not involving the SNAP-25 cleavage are extremely relevant to the ubiquitous therapeutic potential of BoNT/A. Numerous in vitro and in vivo studies indicate that the actions of BoNT/A are linked to processes such as the cell cycle, apoptosis, neuritogenesis, and gene expression. Thanks to its high potency, prolonged effects, and reliable safety profile, BoNT is empirically utilized across various fields, including ophthalmology, gastroenterology, urology, orthopedics, and dermatology, as well as in treating secretory issues and pain disorders. However, available and approved BoNT therapies are exclusively focused on neuronal territories, with periodic injections often causing immunoresistance for a wide range of indications. The engineering of novel BoNTs will support the development of non-neuronal therapies and facilitate the reduction of immunoresistance. As a result, many studies need to address the underlying mechanisms leading to the very wide therapeutic indications of BoNTs from a mechanistic and therapeutic perspective [19].

In addition, BoNT/A action at the neuromuscular junction exerts prolonged inhibitory effects on neurotransmission, but no quantitative analysis has been conducted to establish dose-related effects. At low calcium concentrations, BoNT/A completely blocked K+-stimulated release but not at higher calcium concentrations, showing that a pool of neurotransmitter can be released after complete cleavage of SNAP-25. Those findings confirm that partial neurotransmission can persist without SNAP-25, suggesting that full blockade by BoNT/A may be achieved through additional targets [20,21].

### 2.1. Effects on Gene Expression

#### 2.1.1. In Vivo Studies

BoNT/A injections reduce neuropathic pain-related behaviors in rats through the modulation of the activation of spinal microglia. BoNT/A inhibits injury-activated neuronal function in structures distant from the injection site, increasing mRNA levels of NOS1, prodynorphin, and pronociceptin in the dorsal root ganglia (DRG). This pain relief effect may be related to SNAP-25 cleavage, but no study has yet been performed to identify the influence of SNAP-25 cleavage in this action on neuropathic pain [22] (Figure 1). More recently, BoNT/A has been found to restore the neuroimmune balance between pronociceptive (IL-1β and IL-18) and antinociceptive (IL-10 and IL-1RA) factors in neuropathic rats [23] through the TLR2-dependent pathway in macrophages [24]. In line with precursor studies on the modulation of inflammation, subsequent studies showed that BoNT/A suppresses the expression of pro-inflammatory factors by modulating NF-κB, p38, and ERK1/2 pathways, while simultaneously enhancing the expression of TLR2 and its adaptor protein, MyD88, in microglial cells [25] (Figure 1).

#### 2.1.2. Effects in Cell Models

The molecular effects of BoNTs on intoxicated neurons in parallel to the cleavage of SNAREs have not been explored in detail. A microarray analysis was conducted to examine the influence of BoNT/A and its atoxic derivative (BoNT/A ad) on the transcriptome of neurons derived from human induced pluripotent stem cells (hiPSC). This study revealed significant alterations in 492 genes following exposure to BoNT/A and 682 gene alterations for the atoxic derivative, when assessed 2 days and 2 weeks post-exposure, compared to populations that were not exposed. The most highly regulated genes in cells exposed to BoNT/A1 or BoNT/A are related to neurite outgrowth and calcium channel sensitization, demonstrating that BoNT/A exerts a myriad of molecular effects in addition to the cleavage of SNAREs [26]. In line with those findings, a massive host response was also evidenced in SH-SY5Y neuronal cells involving the modulation of neuroinflammatory, ubiquitin-proteasome degradation, phosphatidylinositol pathways, and calcium signaling, and genes relevant to focal adhesion, cell adhesion molecules, adherens, and gap junction-related pathways in HT-29 cells, which is suggestive of a profound modulation of gene expression during exposure to BoNT/A in those two cell types. A differential response was found between epithelial and neuronal cells, showing a distinct role in BoNTs’ cellular mode of action in different host cell types [27].

### 2.2. Targeting the Rho Pathway

The inhibitory effect of botulinum neurotoxins (BoNTs) is primarily due to their metalloprotease activity on the SNAP-25 protein, vital for the exocytotic machinery. However, the cleavage of SNAP-25 does not entirely block neuroexocytosis, suggesting that botulinum’s inhibition involves a more intricate mechanism. Rho GTPases play a pivotal role in modulating the organization of the actin cytoskeleton, thereby governing numerous cellular functions, with significant implications for neuronal cell activity. Specifically, the RhoB signaling pathway influences acetylcholine release through mechanisms involving the reorganization of the actin cytoskeleton. The authors [28] demonstrated that the G protein activator lysophosphatidic acid (LPA) initiates actin reorganization and subsequently induces Ca^2+^-dependent acetylcholine (ACh) release in PC12 cells treated with nerve growth factor. They found that BoNT/A disrupts both processes by degrading RhoB via the proteasome. This led them to conclude that botulinum toxin inhibits neuroexocytosis by also targeting the RhoB pathway [28].

### 2.3. Effects on Cancer Cells

An excellent review [29] about the effects of BoNT/A on a range of cancer cells was previously published. This systematic review, based on 15 articles on neoplastic cell lines and eight articles on in vivo neoplasms, suggests that the botulinum toxin disrupts the progression of cancer cells, including apoptotic effects. Of note, no safety issues were reported during in vivo studies [29].

#### 2.3.1. Effects on Cancer Cell Lines

BoNT/A is able to inhibit the growth of LNCaP human prostate cancer cells in vitro and in vivo, inducing apoptosis in a dose-dependent manner [30,31]. BoNT/A also induces the up-regulation of 167 genes and down-regulation of 60 genes involved in focal adhesion, cell adhesion molecules, and gap junction pathways within carcinoma cell lines, showing a wide action on cancer cell proliferation [27,32,33]. The effects of BoNT/A on cell proliferation and apoptosis were also tested on the T47D human breast cancer cell line. Botulinum toxin A exhibited a greater cytotoxic activity in T47D cells compared with MCF10A normal cells by eliciting caspase-3 and -7 dependent apoptotic processes in the T47D breast cancer cell line [34]. In addition, a mathematical model was developed to quantify the tumor-suppressing effect of BoNT/A1 in B16-F10 melanoma cancer cells [35].

#### 2.3.2. Effects on In Vivo Cancer Models

The synergistic antitumor effects of BoNT/A1 combined with PD-1 checkpoint blockade were demonstrated in mouse in vivo models of melanoma and colon carcinoma. This highlights the potential of BoNT/A1 as an anti-cancer treatment when used alongside immune checkpoint blockade therapy [36].

The modulations of genes in different cell types are listed in Table 1 below.

The complex interactions between cancer-related neurons and the communication networks at the nerve–cancer interface continue to remain inadequately understood. Despite this, experimental models of cancer denervation conducted in vivo using rodents, as well as human models, have underscored a significant dependency of cancer cells on neuronal inputs. Despite this, the fundamental mechanisms driving nerve-mediated cancer aggressiveness are still not fully understood. Experimental studies have revealed that cancer cells with mitochondria transferred from neurons exhibit increased metastatic potential. This finding offers new insights into the impact of the nervous system on cancer metabolism and the spread of metastasis [38]. The study of human cancer cells evidenced essential interactions between human cancer and nerves for disease progression. A similar denervation profile was observed in tumors of denervated rodent prostates, in spinal cord injury patients, and in Botox-treated patients. Denervation also indicated translation and bioenergetic shutdown due to basic cellular deregulation of non-neoplastic epithelial cells. Those findings will significantly influence general cancer treatment strategies, as targeting the neural microenvironment offers a therapeutic approach for treating human prostate cancer [39].

### 2.4. Interaction with the Fibroblast Growth Factor Receptor

More recently, the fibroblast growth factor receptor (FGFR3) has been discovered as contributing to the specific uptake of BoNT/A into presynaptic nerve terminals and being involved in a large receptor complex formation, including gangliosides and protein interactions. The formation of this receptor complex is tightly regulated through the combination of high- and low-affinity receptors. This complex receptor-binding process suggests that the actions of BoNTs once internalized into neuronal cells are not limited to the SNARE protein cleavage and involve additional targets [40].

### 2.5. Effects on Secretions and Inflammation

The involvement of arachidonic acid (AA) in BoNT intoxication was described in early studies. The membrane depolarization leads to increased intraterminal Ca^2+^ concentration subsequent to voltage-sensitive calcium channel activation, which leads to phospholipase activation, triggering AA release from membrane stores. This biochemical sequence underlines the role of AA in the BoNT intoxication process. The ability to block the release of AA in this PC12 cell line model provides a biochemical process underlying the anti-inflammatory effects of BoNT [41]. Further studies established that PLA(2) and AA have important roles in neuroexocytosis independent of SNAP-25. Both are involved in organizing the actin cytoskeleton, which is essential for vesicle fusion and exocytosis. This mechanism serves as an alternative molecular target for BoNT/A [42]. In line with those early studies, mastoparan delivered into BoNT/A intoxicated spinal cord cells restored 40% of stimulated neurotransmitter release. The rescue from cell poisoning was not related to inhibition of the activity of BoNT/A light chain (LC) against its SNAP-25 substrate, but instead, Mas-7 induced a physiological host response related to the phospholipase-mediated signal transduction pathway, directly showing the implication of BoNT/A in this pathway [43]. Subsequent studies on plausible actions beyond SNAP-25 explored the trafficking of H^+^-ATPase vesicles to the apical membrane of inner medullary collecting duct cells, utilizing a mechanism similar to the one found in the neurosecretory cells involving the formation of SNARE complexes. The SNAP-23 complex was identified as a new substrate for botulinum toxin proteolysis, having a critical role in the regulation of H^+^-ATPase exocytosis and H^+^ secretion in renal epithelial cells [44].

### 2.6. Effects on Intestinal Peristalsis and Microbiota Composition

In vivo studies in mice have recently revealed that the release of subclinical doses of BoNT/A and BoNT B into the intestinal lumen induces enteric neuron paralysis while facilitating infection by bacterial pathogens. The BoNTs cause alterations in the release of mucin, subsequently leading to a disruption of the protective barrier against intestinal infections. These seminal findings highlight the role of BoNTs in intestinal peristalsis and infections, establishing for the first time the link between botulinum toxins, enteric neuron function, and intestinal immune system performance. The interaction between the nervous and immune systems plays a pivotal role in regulating the composition of intestinal microbiota by preventing the invasion of bacteria, fungi, and parasites. However, the current detection methods lack the sensitivity required to identify circulating botulinum neurotoxin (BoNT) within the enteric system. Nevertheless, those findings open the possibility that BoNTs may indirectly attenuate local chronic pathological inflammations, although further investigations are needed to establish the effects on immune cells [45].

## 3. Effects on Dermal Fibroblasts and Regeneration

Despite the wide range of treatments currently available for tissue repair and regeneration, there is a pressing need for innovative therapies to enhance patients’ overall quality of life. In recent years, the application of BoNT/A has provided promising results in tissue repair and regeneration [46]. In vitro studies on human fibroblasts showed the beneficial effects of BoNT on collagen production, and this was confirmed by intradermal BoNT injection for remodeling dermal tissues in aged skin [47].

### 3.1. Reduction in Hypertrophic Scars

Since its approval for cosmetic applications, the safety and efficacy of botulinum toxin type A to treat traumatic or postoperative pathological scars have become a strong research focus, allowing dermatologists to evidence several options to improve wound healing and postoperative scar appearance [48].

#### 3.1.1. Effects on Fibroblasts Derived from Hypertrophic Scars

In addition to the beneficial effects on aged skin, BoNT/A effectively inhibits the growth of hypertrophic scar-derived fibroblasts with a concomitant decrease in connective tissue growth factor (CTGF), adding a synergistic effect in reducing hypertrophic scarring [49]. Clinical observations confirmed that BoNT/A can inhibit growth and improve hypertrophic scar appearance. To complete clinical observations, the cell cycle distribution was evidenced as a potential molecular mechanism explaining the clinical benefit of BoNT/A [50]. However, the precise mechanisms underpinning hypertrophic scars and keloids are still not fully elucidated.

#### 3.1.2. Effects on Hypertrophic Scars in the Clinic

The effects of BoNT/A on wound tension, collagen, and fibroblasts have been extensively explored in a systematic literature search resulting in 1000 articles. A total of 35 articles that satisfied the selection criteria have been identified, elucidating the role and mechanisms of action of botulinum toxin type A in both the treatment and prevention of hypertrophic scars and keloids. These studies contribute to understanding the mechanism of action of botulinum toxin type A [51].

Botulinum toxin A is a favorable treatment for hypertrophic and keloid scars, as evidenced in the clinic. The primary hypothesis resides in the reduction in wound edge tension subsequent to the relaxation of supporting musculature. However, it soon appeared that the mechanisms of action are indeed much more complex since BoNT-A also affects fibroblast function and collagen production. Botulinum toxin type A (BoNT-A) is impacting the expression of transforming growth factor-B1, myosin II, alpha-smooth muscle actin (α-SMA), and connective tissue growth factor (CTGF). Simultaneously, it is associated with a reduction in inflammation and an increase in phosphatase and tensin homolog (PTEN) expression [52].

A variety of treatments are currently available to reduce hypertrophic scars, but they provide modest therapeutic results. A preliminary study suggested that BoNT/A significantly reduced the growth of fibroblasts derived from hypertrophic scar in parallel with a decrease in TGF-β1 protein [53]. In parallel to its effects on collagen, BoNT/A causes a decrease in the connective tissue growth factor (a regulator of TGF-β1 function responsible for scarring and fibrosis), confirming its ability to control hypertrophic scarring [49]. Recent studies show the efficiency of BoNT/A in alleviating scar contracture (SC) resulting from major burn injuries. The proliferation of fibroblasts was significantly inhibited by BoNT/A, showing that BoNT/A inhibits the growth of fibroblasts derived from SC and reduces the expression of α-SMA and myosin II [54].

In addition to the effects on collagen production, botulinum toxin A reduces skin tension in wound sites and scar hyperplasia. A comprehensive study spanning four electronic databases evaluated several scales, including the Visual Analogue Scale (VAS), Vancouver Scar Scale (VSS), scar width, the Patient Self-Assessment Scale, Stony Brook Scar Evaluation Scale, Observer Scar Assessment Scale, Manchester Scar Scale, and Patient Scar-Assessment Scale. This systematic review incorporated 20 studies, involving a total of 894 patients. The findings demonstrated that both the VAS and VSS showed significant improvement with the use of BTA, which also contributed to a reduction in scar width and enhanced outcomes for the upper lip and forehead [55]. A different meta-analysis sought to assess the effectiveness of BNT/A in preventing the excessive growth of postoperative facial scars and enhancing scar quality. By employing the Vancouver Scar Scale (VSS), Visual Analogue Scale (VAS), Observer Scar Assessment Scale (OSAS), Patient Scar Assessment Scale (PSAS), and Stony Brook Scar Evaluation Scale (SBSES), the study indicated that complications showed a higher VAS score, lower VSS and OSAS scores, and reduced scar width with the use of BoNT/A. This meta-analysis confirmed that BoNT/A improves the appearance of postoperative facial scars by significantly inhibiting scar hyperplasia and improving scar quality [56].

### 3.2. Effects in the Treatment of Keloids Through Collagen Modulation

In order to further explore the effects of BoNT on human dermal fibroblasts in vitro, the measurement of the procollagen type I carboxy-terminal peptide (PIP) was conducted using an enzyme-linked immunosorbent assay. Additionally, collagen production was tracked through Western blotting. The results showed a significant increase in PIP levels when BoNT was present, and BoNT also enhanced the expression of type I collagen. This study provided experimental evidence of the beneficial effects of intradermal BoNT in the remodeling of dermal tissues in aged skin based on PIP levels monitoring [47]. The study delved into the therapeutic impact of BoNT on keloids by examining mRNA expressions of collagen types I and III, along with MMP-1 and TIMP-1. The findings revealed that type III collagen mRNA expression decreased after administering BoNT/A, whereas MMP-1 mRNA expression progressively increased with higher doses of BoNT/A. The down-regulation of collagen III expression, up-regulation of MMP-1, and increased MMP-2 enzymatic activity in keloid fibroblasts after BoNT/A administration directly reduced the excess collagen deposition in keloids [57]. In addition to the effects on collagen, expression levels of S100A4 (S100A4 gene), TGF-β1 (transforming growth factor), VEGF (vascular endothelial growth factor), MMP-1 (matrix metalloproteinase-1), and PDGFA (platelet-derived growth factor) genes were found to be altered in keloid fibroblasts treated with BoNT/A, supporting the beneficial role of BoNT-A as a novel treatment for keloid scarring [58].

### 3.3. Benefits of Botulinum Toxin A in Tissue Repair and Regeneration

#### 3.3.1. Benefits of BoNT/A in Silicone-Induced Capsule Formation

Capsular contracture represents one of the most serious complications of post-breast surgery, but the mechanism of capsule formation has not been fully clarified. Botulinum toxin type A treatment in vivo reduced capsule thickness and alpha-smooth muscle actin (Smad2) phosphorylation in silicone-induced capsules, confirming the beneficial role of BoNT/A against capsule formation [59]. Studies conducted in mice have demonstrated that BoNT-A effectively inhibits the formation of capsules around silicone implants. This is achieved by blocking TGF-β1 signaling and preventing fibroblasts from differentiating into myofibroblasts [60].

#### 3.3.2. Benefits in Skin Wound Healing and Engraftment

In addition to its effects on TGF-β signaling and myofibroblast differentiation, investigations on the effects of BoNT/A on macrophage polarization during skin repair established that application of BoNT/A significantly reduced the scar diameter in parallel with the improvement in fiber proliferation and inhibition of angiogenesis in scar granular tissue. BoNT/A induced a reduction in M1 macrophage numbers and pro-inflammatory factors such as LPS-activated JAK2/STAT1 (Janus kinase/signal transducer and activator of transcription) and IκB/NFκB (inhibitor of nuclear factor kappa B/Nuclear factor kappa B) pathways, which contribute to scar alleviation [61]. BoNT/A enhanced the long-term weight and volume retention of adipose tissue grafts in rat models while maintaining cellular integrity. It notably elevated the expression levels of CD31 and vascular endothelial growth factor (VEGF), indicating its role in promoting vasodilation and the proliferation of endothelial cells. In addition, BoNT/A increased adipose-derived stem cell proliferation and adipogenic differentiation capacity, as well as the expression level of the key cytokines supporting angiogenesis. On the whole, BoNTA was found to facilitate adipose tissue engraftment as well as adipose stem cell regeneration, which could be beneficial in future clinical applications [62].

#### 3.3.3. Combination Approach: Microneedling Combined with BoNT

The effectiveness of incobotulinumtoxinA in combination with microneedling was assessed on postoperative facial scars in subjects undergoing surgical removal of facial non-melanoma skin cancers followed by flap reconstruction. The use of incobotulinumtoxinA improved scar appearance, significantly reduced Vancouver Scar Scale scores, and improved the overall satisfaction of treated subjects when combined with microneedling [63]. Treatment with microneedling and platelet-rich plasma of subjects with atrophic post-acne scars was compared with a microneedling and Botox combination. Both combinations improved the acne scars with comparable efficacy [64].

### 3.4. Reduction in Skin Inflammation

#### 3.4.1. Experimental Models

In addition to benefits in scar reduction and tissue repair, BoNT/ A and B can reduce skin inflammation by inhibiting degranulation of both human and murine mast cells. The presence of SNAREs on mast cells suggests that BoNT’s action can extend beyond the regulation of neuronal activity. The introduction of BoNT/A considerably diminished skin redness, mast cell degranulation, and the mRNA expression of biomarkers linked to rosacea. These results indicate that the toxin helps reduce skin inflammation associated with rosacea by directly preventing mast cell degranulation and deserves further study to clarify its mechanism of action and therapeutic potential in this area [65].

#### 3.4.2. Reduction in Skin Inflammation in the Clinic

Similarly, recent clinical research evidenced the beneficial effects of a single dose of BoNT/E in patients with itch and pain. In comparison to a placebo, a single dose of 2.8 ng BoNT/E was safe and effectively reduced itch and pain. However, it did not have an impact on wound healing or scar formation [66].

The study demonstrated that two serotypes of BoNT showed prolonged anti-itch effects in a mouse model of itching induced by two different pruritogens, each working through distinct mechanisms. The findings also support the idea that BoNTs might directly influence mast cell degranulation [67].

The effects of BoNTs on skin regeneration are described in Table 2 below.

## 4. Effects on Pain, Neuropathy and Epilepsy

In the seminal review by Matak and Lackovic in 2014 [68], the relevant mechanisms underlying BoNT/A antinociceptive actions were revisited in detail. Initially deemed to be of peripheral origin [69,70,71], subsequent experiments documented the axonal transport of BoNT/A into central sensory regions, supporting centrally mediated antinociceptive effects [72,73,74]. This review sparked many additional questions on the mechanism of BoNT/A antinociceptive action and other effects, which still need to be further explored today. The effect of BoNT at the neuromuscular junction is classically known to be due to the inhibition of acetylcholine release; however, this effect alone cannot justify the wide range of analgesic activity of BoNT. Scientific and clinical evidence suggest an array of antinociceptive mechanisms for botulinum toxins in chronic musculoskeletal, neurological, pelvic, perineal, osteoarticular, and headache conditions [71]. Early on, it became evident that BoNT/A provided a long-lasting analgesic effect in pain related to movement disorders. Subsequent observations of its effectiveness in treating neuropathic pain and headaches further validated that BoNT/A’s pain-relieving properties are independent of its anticholinergic effects. While chronic migraine remains the sole officially approved pain-related use for BoNT/A, its effectiveness has also been demonstrated in managing chronic pain conditions such as trigeminal neuralgia, postherpetic neuralgia, and posttraumatic neuralgia, and also for diabetic neuropathy, plantar fasciitis, piriformis syndrome, pain from total knee replacement, male pelvic pain syndrome, chronic low back pain, and neuropathic pain resulting from traumatic spinal cord injury [75].

### 4.1. Suppression of the Substance P/NK1R Pathway

The role of substance P (SP) and NK1R was explored in the antinociceptive activity of BoNT/ A in vivo. BoNT/A activity was prevented by the deletion of SP/ NKA and NK1R genes in acute and chronic inflammatory pain, as well as neuropathic pain, in mice, suggesting that the analgesic effect of BoNT/A was related to the SP/NK1R-pathway integrity. This study evidenced the direct role of BoNT/A in the inhibition of neuronal activation and c-Fos expression in the dorsal horn of the mice [76]. There is currently no therapy for “mirror pain” (MP), which is pain opposite to the side of injury, and the mechanism is not yet clarified in humans. In line with the studies documenting axonal transport of BoNT/A, the unilateral injection of BoNT/A has a bilateral effect in experimental MP, showing BoNT/A as a potential therapy for this condition. In vivo experiments confirmed the segmental actions of BoNT/A at the spinal level [77]. In addition, bilateral analgesic effects of aboBoNT-A were evidenced following unilateral administration in more than one model of polyneuropathy and chronic inflammatory pain (rendered bilateral). The mechanism responsible for the bilateral analgesic effects of aboBoNT-A likely involves the activation of peripheral sensory neurons and may also include the retrograde axonal transport of the toxin into the spinal cord [78].

Studies by Lackovic and coworkers also established the influence of BoNT/A on thermal hyperalgesia, mechanical hyperalgesia, and carrageenan-induced local edema formation. BoNT/A reduced capsaicin- and carrageenan-induced pain but not carrageenan-induced edema. However, considering the several-day delay in the onset of antinociceptive activity of BoNT/A, as well as its effectiveness in acute and chronic experimental pain, but its lack of effect on paw edema, the authors suggested for the first time that the mechanism of the antinociceptive action of BoNT/A was more complex than the exclusive inhibition of neurotransmitter release [79].

### 4.2. Downregulation of TRPV1 and TRPA1

BoNT/A selectivity for certain types of pain is also related to capsaicin-sensitive neurons. Presynaptic activity of BoNT/A is evidenced in TRPV1-expressing neurons by localization of cleaved SNAP-25. In addition, BoNT/A reduced c-Fos activation, suggesting a reduction in nociceptive transmission at the central synapse of primary afferents using different mechanisms, including targeting of TRPV1-expressing neurons, thus explaining its action on certain types of pain [80].

The extension of the use of BoNTs to the central nervous system (CNS) led to the study of their traffic in central neurons and the role of SNAP-25 cleavage. Fluorescent BoNT/A and BoNT/E were used to study their penetration, translocation, and catalytic action into excitatory and inhibitory neurons. In addition to their preferential targeting of glutamatergic neurons, BoNT/A and BoNT/E are more efficient in impairing the release of excitatory than inhibitory neurotransmitters. However, it is plausible that BoNT/A or /E affects substrates beyond SNAP-25, which is supported by evidence of BoNT/A activity occurring without its usual proteolytic target, SNAP-25. Specifically, BoNT/A reduces glutamate release from cultured astrocytes, even though these cells do not express SNAP-25. Additionally, BoNT/A hampers the activation of store-operated calcium currents in cell types such as oocytes and HEK293, where SNAP-25 is not present [81].

Empirical investigations conducted both in vivo and in vitro have demonstrated that botulinum neurotoxin type A (BoNT/A) obstructs the recruitment of Transient Receptor Potential Vanilloid 1 (TRPV1) to the plasma membrane. This disruption consequently results in the intracellular degradation of TRPV1 via the proteasome pathway. Furthermore, behavioral analyses indicate that the antinociceptive effects of BoNT/A exhibit a selective action towards pain mediated by TRPV1 [82]. BoNT/A induces a long-term anti-itch effect by triggering downregulation of TRPV1 and TRPA1 in the dorsal root ganglia in mice. It was hypothesized that the downregulation of the expression of TRPA1 and TRPV1 is contributing to the long-term anti-itch effects of BoNT/A in mice that may serve as an alternative anti-itch therapy [83].

### 4.3. Reduction in Arthritic Pain Through the Inhibition of Nociceptive Peptides and Suppression of Cytokine Expression

Similarly, botulinum toxin type A reduces TRPV1 expression in vivo in the dorsal root ganglion in rats with adjuvant-arthritis pain [84]. The administration of BoNT/A showed promising efficacy in arthritic pain in clinical studies, but further investigations are needed to clarify the underlying mechanism of BoNT/A. In the arthritic rat model, the mechanism is mediated by the inhibition of nociceptive peptides released at the injection site and the suppression of expression of inflammatory cytokines [85]. The antinociceptive effects of BoNT/A were demonstrated in vivo and in clinical settings. It was found that the deletion of SP/NKA and NK1R genes prevented the BoNT/A antinociceptive activity in acute and chronic inflammatory pain and in neuropathic pain. This study confirmed the role of SP-NK1R signaling in the antinociceptive action of BoNT/A [76]. Pretreatment with BoNT/A, alone or in combination with gabapentin, reduced the postoperative pain and inhibited spinal substance P release in vivo. In addition to the SNAP-25 cleavage, the mechanism of action of BoNT/A involved the reduction in NK1 receptor internalization in rats, and subsequently, a decrease in substance P release from the central terminals [86].

### 4.4. Role of Botulinum in Neuropathy

As shown in previous studies on pain, the analgesic effect of BoNT/A was confirmed in vivo in a neuropathy (NP) model in the rat. BoNT/A promoted microglial M2 polarization and reduced neuropathic pain through the reduction in P2X7 receptor density. In this NP model, the mechanical withdrawal threshold and thermal withdrawal latency values in NP model rats were prolonged after BoNT/A administration [87]. BoNT/A has been found to alleviate neuropathic pain caused by chronic constriction injury to the sciatic nerve in both mice and rats. The pain-relieving effect begins 24 h after BoNT/A administration and persists for a considerable time. In mice, a higher dose (15 pg per paw) demonstrates this effect for up to 81 days, while in rats, it lasts 25 days with 75 pg per paw and 35 days after intrathecal injection (75 pg per rat). This model reveals that BoNT/A provides prolonged relief from allodynia and hyperalgesia in animal models of neuropathic pain and supports faster nerve regeneration, as evidenced by behavioral assessments and immunohistochemistry/blotting studies [88].

In vivo studies in mice also show that BoNT/B can reduce neuropathic pain similarly to BoNT/A over a long period of time. However, the analgesic effects do not lead to improved functional recovery, underscoring a significant distinction between the two serotypes in the long-term management of chronic pain [89].

The different biological markers associated with peripheral nerve injury are presented in Table 3 below.

In summary, the use of botulinum neurotoxins (BoNTs) in the treatment of motor and autonomic disorders has been recently described in numerous studies. Besides its action on specific sites of the SNARE protein complex on the peripheral neuromuscular junction (NMJ), BoNT interferes with neural transmission, influencing the release of other neurotransmitters such as glutamate, substance P, and CGRP, but also with glial cells. Recent comprehensive updates on experimental data from animal studies reveal direct interactions between BoNTs and glial cells. These interactions show that BoNTs can inhibit the activation of spinal glial cells, including the release of pro-inflammatory factors. Additionally, BoNTs have the capability to enhance peripheral regeneration by stimulating the proliferation of Schwann cells [90].

Diabetic neuropathic pain is a complication that affects 15–20% of individuals with diabetes, leading to depression, poor sleep, and a lower quality of life. Existing pharmacological treatments often fail to offer adequate efficacy or safety. Subcutaneous or intradermal BoNT/A is an effective and safe treatment for neuropathic pain but is limited in terms of treating the affected area with injections. However, infiltration of the sensory nerve supply with BoNT/A could provide similar effects [91].

### 4.5. Effects of BoNT in the Treatment of Focal Dystonia and Musician Dystonia

Many patients treated with BoNT report improvement in their dystonia without muscle weakness or maintain improvement for a long time after the recovery of weakness. Considering the dissociation between muscle weakness and dystonia relief, it was hypothesized that other effects largely contribute to its clinical benefits. Botulinum toxin (BoNT) may indirectly influence the cortex and spinal cord through afferent pathways. Studies involving electromyography (EMG) and transcranial magnetic stimulation (TMS) have shown a connection between BoNT use and its effects in treating focal dystonia. These effects include a reduction in reflexes and evoked potentials, which are linked to cortical activity and plasticity. Additionally, motor-evoked potentials tend to decrease following BoNT treatment, and this decrease is associated with improvements in clinical dystonia scores. Furthermore, BoNT may alleviate dystonic pain by inhibiting the release of substance P, glutamate, and the calcitonin gene-related peptide [92].

In summary, BoNT/A injections should be included in routine therapy designed for the management of focal task-specific dystonia, more specifically for patients experiencing side effects or lack of benefits of oral pharmacologic agents. In the treatment of musicians with dystonic movements, the clinician has to target specific muscles of interest through precise localization and injection schemes in parallel with the evaluation of limb movement during instrument performance. There is increasing evidence showing the peripheral effects of BoNT at gamma motor neurons, reducing afferent sensory input from muscle spindles to the central nervous system, and influencing sensorimotor pathways [93].

## 5. Neurogenic Actions of Botulinum Toxins

As suggested above by Luvisetto and collaborators, BoNTs can contribute to neuronal regeneration. In early studies, in vivo injection of BoNT/D neurotoxin triggered the outgrowth of the motor nerve from the terminal arborization of the mouse levator auris muscle subsequent to its paralysis. Approximately 25 to 30 days following poisoning, there was a noted recovery in the functionality of the affected junctions. This recovery was observed in conjunction with a synchronized release of quantal transmitters when nerve stimulation occurred, which in turn triggered action potentials and muscle contraction [94]. In addition, the activity of caspases, recognized markers of apoptosis, is substantially reduced by hemaglutinin-33 (HN-33), as well as the degradation of chromosomal DNA in SH-SY5Y cells incubated with HN-33. Purified BoNT/A induced marked anti-apoptotic activity in the same cell model, demonstrating the potential to use BoNT as a therapeutic agent in apoptosis [95].

In addition to its effects on pain, the ability of BoNT/A to induce nerve outgrowth, or sprouting, is classically recognized as a direct consequence of its paralytic actions, but the factors initiating this sprouting have not been identified. The binding of BoNT/A to the motor neuronal membrane triggers neuritogenesis independently of its effects on vesicular release, but the exact intracellular mechanisms remain to be clarified [96]. Experimental studies in the last century have already recognized the nerve growth following botulinum toxin injection as a form of regeneration to compensate for loss of function within the nerve terminal, but without specific lack of structural loss. Considering that motor nerve growth does not normally occur once neuromuscular connections are formed, the restoration of contact within the muscle fiber suggests that a secondary inhibitory mechanism is usually activated to control the formation of new connections [97].

Many peripheral nerve injuries result in ongoing neuropathic pain and partial functional recovery. Recent animal studies have shown the positive impact of botulinum neurotoxin A (BoNT/A) on nerve regeneration and functional recovery following peripheral nerve injury. Proposed mechanisms of its action involve the activation of support cells such as Schwann cells, mast cells, and macrophages, as well as enhancements in angiogenesis and blood flow that aid in nerve regeneration [98].

### 5.1. BoNT Intoxication Induces Nerve Sprouting

Evaluation of the effects of in vivo injection of BoNT/A toxin on endplates of the mouse levator auris longus muscle revealed sprouts of unmyelinated fibers parallel to the longitudinal axis of the muscle fibers with accumulation of acetylcholine receptors. Electrode recordings showed widespread triphasic waveforms, indicating impulse propagation of action potentials alongside the poisoned endings. Those findings confirmed the essential role of the terminal sprouts in the recovery of neuromuscular transmission after BoNT poisoning [99]. Early studies using in vivo imaging within nerve endings of the mouse sternomastoid muscle revealed the inhibition of stimulated uptake of the dye at the terminals within a few days after injection of BoNT/A, in parallel to an increase in staining of newly formed sprouts. After 28 days, vesicle recycling occurred only within the newly connected sprouts and not in parent terminals. However, a second phase of the rehabilitation process followed with a return of vesicle turnover from original terminals in parallel to the elimination of superfluous sprouts. This removal of new sprouts indicates their fundamental importance in the repair of paralyzed endplates with implication for nerve regeneration [100]. Quantitative analysis of endplates from BoNT-A-poisoned muscles of the frog also revealed a higher and significant incidence of terminal and ultraterminal sprouts in poisoned junctions, showing that prolonged neuromuscular blockade by BoNT/A induced significant extension of the nerve terminal arborization [101].

Researchers suggested that BoNT/A promotes neurite growth via presynaptic receptor activation, separate from its intracellular action of cleaving SNAP-25. This was examined in vitro using primary cultures enriched with embryonic mouse spinal cord motor neurons. At concentrations as low as 0.01 nM, BoNT/A significantly enhanced neuritogenesis, an effect that was countered by Triticum vulgaris lectin, a known competitive antagonist of BoNT. The study demonstrated that BoNT/A could initiate neurite growth simply through its binding to the neurons, without requiring SNAP-25 cleavage. The findings suggest that BoNT/A’s attachment to the motor neuronal membrane triggers neuritogenesis through yet unidentified intracellular pathways that are distinct from its paralytic effects [96].

### 5.2. Evidence of Beneficial Effects in Experimental Models of Nerve Injury

The study examined the expression levels of several peptides, including enkephalin (ENK), acidic fibroblast growth factor (aFGF), neurotensin (NT), galanin (GAL), substance P (SP), vasoactive intestinal polypeptide (VIP), and neuropeptide Y (NPY), in the spinal motoneurons of rats following chemodenervation of the gastrocnemius muscle using BoNT/A treatment. ENK expression was notably upregulated not only in the region of the affected motoneurons but also on the opposite side and in more remote areas of the spinal cord. The peak in ENK expression was observed between 7 and 14 days post-injection, followed by a gradual decline over a 3-month period. These findings give demonstration of the plasticity in genetic expression regulation of the adult spinal cord following botulinum toxin treatment [102].

After 8 days, aFGF was slightly reduced in all the regions examined. Single motoneurons exhibited NT expression, but there was no detectable expression of GAL, SP, VIP, or NPY in either the control or toxin-treated neurons. This study is an additional demonstration of the gene adaptation and plasticity of spinal cord neurons following botulinum toxin treatment as a first indication of the underlying mechanisms.

Botulinum neurotoxin A (BoNT) and brain-derived neurotrophic factor (BDNF) influence synaptic inputs to neurons and modulate deafferentation of motoneurons following nerve section, improving peripheral nerve regeneration. A significant reduction in the loss of perisomatic cholinergic terminals was observed after BoNT injection at 1 week and 2 months post-injury. The intraneural application of BoNT represents a promising treatment of deafferentation, revealing new insights into synaptic plasticity and restoration of function [103]. Using retrograde tracers in parallel with histological analysis and immunochemistry, BoNT treatment significantly enhanced regeneration of the murine motor axons in vivo and in human embryonic stem cells in vitro. The preconditioning with BoNT/A was identified as a suitable treatment approach to enhance motor axon regeneration in specific clinical situations requiring nerve transfer surgery, but further studies are also needed to elucidate the molecular mechanism [104].

The effects of botulinum neurotoxin A on nerve regeneration were also assessed in the rat with sciatic nerve injury. The groups receiving a single dose of intraneural BoNT/A immediately after nerve injury exhibited a higher expression of GFAP, S100β, GAP43, NF200, BDNF, and NGF whatever the BoNT/A dose group. In parallel, average area and myelin thickness, as well as sciatic functional index and compound muscle action potential amplitudes, were significantly greater in the BoNT/A groups. These findings confirmed the beneficial effects of BoNT/A in nerve regeneration [105]. The effects of intranerve application of BoNT/A were also assessed on regeneration and functional recovery following peripheral nerve injuries in the mouse. BoNT/A improved the axonal regeneration rate by inducing longer nerve profiles, as confirmed using nerve histology. BoNT/A enhanced the reinnervation of gastrocnemius and plantar muscles. It was concluded that a low dose of BoNT/A improves sensorimotor recovery by improving axonal regeneration, showing its beneficial application for peripheral neuropathies [106].

Recent research indicates that Ras-related C3 botulinum toxin substrate 1 (Rac1) plays a crucial role in promoting axonal regeneration after brain injury. It achieves this by encouraging neuronal growth and counteracting inhibitory signals that lead to growth cone collapse. Experimental stroke models have validated the vital importance of Rac1 in axonal regeneration and brain remodeling. Rac1 signaling enhances axonal regeneration and improves functional recovery in experimental models of stroke [107].

In preclinical models, BoNT serotype A (BoNT/A) is able to promote axonal regeneration, in addition to presynaptic vesicle fusion blockade. In hippocampal neurons, the non-toxic C-terminal region of the receptor-binding domain of the BoNT/A heavy chain (HCC/A) plays a role in activating the small GTPase Rac1 and the ERK pathway. This activation enhances axonal growth, the formation of dendritic protrusions, and the release of synaptic vesicles. These findings align with the neurotrophic characteristics of the BoNT/A heavy chain, which are separate from its catalytic function [108].

In addition to its effects after nerve surgery, the roles of botulinum neurotoxin A (BoNT/A) and extracorporeal shock wave therapy (ESWT) were assessed in the regeneration of injured peripheral nerves. Treatment with BoNT/A or ESWT induced a higher expression of GFAP (Glial fibrillary acidic protein) and S100β (soluble protein-100β) in injured nerves, in parallel with a significant increase in GAP43 (growth associated protein 43), ATF3 (activating transcription factor 3), and NF200 (neurofilament 200) levels involved in axonal regeneration and significantly higher than in the control group. Electrophysiological, clinical behavioral, and tissue analyses indicated improved functional recovery through the activation of Schwann cells and axonal regrowth following treatment with BoNT/A or ESWT. It was concluded that both BoNT/A and ESWT effectively stimulated Schwann cell activation, promoting axonal regeneration and enhancing the functional recovery of the injured nerve [109].

### 5.3. Clinical Applications

The exploration of the effects of BoNTs at the neuromuscular junction has set the foundation for the use of these toxins in a wide range of human pathologies due to excessive muscle contractions, but in addition to beneficial effects in pain, recent findings point to the potential efficacy of BoNTs in nerve regeneration after traumatic injury, such as sciatic nerve injuries in the peripheral nervous system and spinal cord injury in the central nervous system [110]. In clinical settings, Xeomin^®^ is a commercially available formulation of botulinum neurotoxin type A (BoNT/A), approved for the treatment of neurological conditions such as blepharospasm, cervical dystonia, limb spasticity, and sialorrhea. A proof-of-concept study of the efficacy of Xeomin^®^ in preclinical spinal cord injury has shown functional improvement in paraplegic mice, but its inherent mechanism is far from being understood, opening new avenues for further research [111]. Similarly, examination of the orbicularis muscle of 10 patients receiving injections of botulinum toxin for 6 weeks to 3 years to treat essential blepharospasm revealed characteristic “sprouting” of the motor axons. In parallel, the orbicularis muscle from five individuals never exposed to botulinum failed to demonstrate these changes, but the mechanism underlying motor nerve sprouting in response to botulinum exposure is not yet elucidated [112].

## 6. Role of BoNT in Neurogenic Bladder Disorders and Interstitial Cystitis

The use of BoNT in urological practice is still in the developmental stages, and full investigations are required to elucidate the mechanisms of action.

Initial clinical studies showed therapeutic effects of BoNT/A intradetrusor injections in patients with refractory idiopathic or neurogenic detrusor overactivity. The impact of BoNT/A on urinary bladder function likely involves several mechanisms. Besides its well-documented ability to inhibit the presynaptic release of acetylcholine from motor nerve endings, growing evidence indicates that BoNT/A may also influence sensory fibers. Biopsies from patients with neurogenic or idiopathic detrusor overactivity showed decreased levels of sensory receptors P2X3 and/or TRPV1 that may contribute to the clinical effect of BoNT/A [113]. Significant accumulation of the radiolabeled BoNT/A was evidenced in the lumbosacral DRG, together with a less significant uptake in the respective spinal cord segments, providing clear evidence of the retrograde transport of BoNT/A to the CNS after bladder injection in rats [114].

Research on the influence of BoNT on dorsal root ganglion neurons in vitro associated with the porcine urinary bladder demonstrated a notable reduction in neurons containing substance P, calcitonin gene-related peptide (CGRP), calbindin, somatostatin, and neuronal nitric oxide synthase (nNOS). This indicates that BoNT affects the chemical coding of bladder sensory neurons [115]. In addition, the chemical coding of sympathetic chain ganglia neurons connected to the urinary bladder was investigated in vivo in both normal pigs and those treated with BoNT/A. This study employed a combination of retrograde tracing and double-labeling immunohistochemistry techniques. This study revealed a profound decrease in the number of noradrenergic neurons expressing NPY (neuropeptide Y), VIP (vasoactive intestinal polypeptide), SOM (somatostatin), and GAL (galanin), as well as an increase in the number of neurons expressing nNOS (neuronal nitric oxide synthase) and L-ENK (Leu-enkephalin) in pigs treated with BoNT [37].

Interstitial cystitis/bladder pain syndrome (IC/BPS) is a long-lasting and challenging suite of urological disorders. It is marked by bladder pain or discomfort, particularly when the bladder is filling, and is often associated with lower urinary tract symptoms (LUTS). Clinical investigative initiatives such as The Multidisciplinary Approach to the Study of Chronic Pelvic Pain (MAPP) have categorized IC/BPS patients into four main groups: bladder-centric, which is further divided into those with Hunner lesion disease (HLD) and those without (IC/BPS), as well as categories for widespread/centralized pain and myofascial pelvic floor tenderness [116]. A diagnosis of interstitial cystitis/bladder pain syndrome (IC/BPS) often coincides with a high occurrence of chronic overlapping pain conditions. These include irritable bowel syndrome (IBS), fibromyalgia (FM), chronic fatigue syndrome (CFS), vulvodynia, migraine headaches, temporomandibular joint dysfunction (TMD), and low back pain [117].

Treatment for patients with IC/BPS is always challenging for urologists. While current guidelines recognize BoNT-A injection as a standard treatment for non-Hunner lesion patients, most of the clinical evidence comes from retrospective, uncontrolled studies. So far, only two randomized, placebo-controlled studies with a small number of participants have been published. Both trigone and diffuse BoNT/A injections have demonstrated effectiveness and safety in treating IC/BPS, although further comparative studies are required. It has been observed that most IC/BPS patients typically experience a return of symptoms between 6 and 9 months after receiving a BoNT/A injection. However, ongoing injections have proven to maintain their therapeutic benefits in long-term follow-ups. To confidently establish BoNT/A as a standard treatment for IC/BPS patients, more extensive randomized, placebo-controlled studies involving larger patient groups are necessary [118].

In a phase II study on women with IC/BPS for more than 6 months and pain for 4 months or longer on the Visual Analogue Scale (VAS) of 0 to 10, patients were treated with BoNT/A 100 U injected into the trigone. The primary study endpoint was the change from baseline pain intensity reported at week 12. The proportion of patients who achieved a 50% or greater reduction in the pain using the Visual Analogue Scale was 60% for BoNT/A vs. 22% for placebo, confirming the efficacy of BoNT/A in this context [119].

In the rat isolated urinary bladder, BoNT/A inhibited the evoked release of CGRP from afferent nerve terminals, suggesting a potential clinical benefit for the treatment of interstitial cystitis or sensory urgency [120]. In another preclinical study in rats, the urodynamic functions were evaluated following intradetrusor injection of BoNT/A and the bladder inhibitory effects of spinal nerve stimulation. Botulinum toxin A (BoNT/A) was found to enhance bladder capacity. However, bladder contractions that occur during bladder filling were reduced by sacral nerve stimulation. Notably, BoNT/A did not impact SNS’s effectiveness in suppressing bladder contractions, even after being injected directly into the detrusor muscle. These findings encourage additional preclinical and clinical research to explore the potential benefits of combining neuromodulation with intradetrusor BoNT/A therapy [121].

The main adverse effects of botulinum toxin A in urological practice are urinary retention and urinary tract infection [122], which are probably related to excessive inhibition of efferent neurotransmission in the urinary bladder.

## 7. Botulinum A Administration in Endometrial Disorders

A suitable endometrial growth is essential for successful embryo implantation and maintaining pregnancy. The effectiveness of administering botulinum toxin A directly into the uterus to enhance endometrial angiogenesis and improve embryo implantation rates has been previously presented. The powerful therapeutic impact on repairing the structure and function of the uterus was demonstrated in a mouse model with thin endometrium, which showed a substantial reduction in endometrial thickness and embryo implantation rates. This study revealed that levels of insulin-like growth factor binding protein-3 (IGFBP3) and active osteopontin (OPN) were notably lower in cases of thin endometrium. However, these levels were almost entirely restored following intrauterine administration of BoNT/A. When the BoNT/A-induced IGFBP3 was neutralized, it led to a suppression of OPN’s proteolytic cleavage, resulting in the endometrial thickness not being restored. This suggests that BoNT/A-induced endometrial regeneration may rely on IGFBP3-dependent proteolytic cleavage of OPN. Intrauterine BoNT/A administration improved the endometrial environment by increasing endometrial receptivity and angiogenesis and relying on the regulatory effect of IGFBP3 on OPN proteolytic cleavage, which suggests BoNT/A as an efficient therapeutic strategy for patients with thin endometrium [123].

## 8. Effects of BoNT on Vessel Regeneration, Blood Flow and Tissue Survival

### 8.1. Vascular Benefits of BoNT Applications

#### 8.1.1. Experimental Studies In Vivo

Besides its various effects on nerve regeneration, the application of BoNT/A can also influence vascular regeneration by preventing the collapse of the peripheral vessels in the cutaneous dorsal skin flap of the rat as well as myo-cutaneous flaps, significantly improving flap survival. Similarly to neuronal regeneration effects, further investigations are in progress to determine the mechanism of action by which BoNT/A attenuates the sympathetic vasoconstriction [124,125].

Beyond the advantages offered by BoNT/A, the administration of BoNT/B in mice notably reduced the development of pressure ulcers following cutaneous ischemia-reperfusion injury. This BoNT/B injection effectively mitigated cutaneous I/R-induced vascular damage, decreased hypoxic areas, and lowered the number of oxidative-damaged and apoptotic cells [126]. The investigation into ischemia-reperfusion injury within the rat musculocutaneous flap demonstrated that preconditioning with local BoNT/A provided substantial protection against such injury. Furthermore, there were elevated expressions of CD34, hypoxia-inducible factor 1 alpha (HIF-1α), nuclear factor-kappaB (NF-kB), and vascular endothelial growth factor (VEGF) observed in the BoNT/A group [127]. One in vivo study demonstrated that pretreatment of rats with BoNT/B significantly reduced changes in vascular diameters and flows compared with the control group, demonstrating the anti-vasospastic effects of botulinum toxin B [128].

#### 8.1.2. In Vitro Evidences

In addition, one in vitro study established that BoNT/A induced a decrease in vasoconstriction of isolated endothelium-intact arteries. The vasoconstriction was significantly inhibited only in the presence of endothelium, showing the implication of endothelium in BoNT/A effects. Of note, BoNT/A exerted its effects on both evoked vasoconstriction and non-evoked vasoconstriction conditions. This research has, for the first time, demonstrated that the effect of BoNT/A is endothelium-dependent and that it induces concentration-dependent vasodilation in arteries with an intact endothelium. Additionally, BoNT/A was found to significantly decrease calcium sensitization in the arteries. However, when inhibitors targeting eNOS (nitric oxide synthase) and sGC (guanylate cyclase) were introduced, they completely negated the vasodilatory effect of BoNT/A. These seminal findings indicated that BoNT/A abolishes arterial vasoconstriction by regulating smooth muscle calcium sensitization but also via the eNOS/sGC/cGMP pathway [129].

The vascular effects of BoNT/A are presented in Figure 2 below.

#### 8.1.3. Clinical Evidence

In addition to the effects on vasoconstriction, the effects of botulinum toxin type A (BoNT/A) combined with Minoxidil were assessed in patients with androgenetic alopecia (AGA). Botulinum toxin A injections (60–70 units) were applied to 30–35 sites on the scalp. The treatment group displayed notable differences in hair growth compared to the control group at the 4-month mark (*p*  <  0.001) and 6-month mark (*p*  =  0.0046) after treatment. This group also experienced significant improvements in quality of life at 4 months (*p*  =  0.0009) and 6 months (*p*  =  0.0099). No adverse reactions were noted following the botulinum toxin injections. When combined with Minoxidil, botulinum toxin A effectively enhances hair growth, improves quality of life, and reduces scalp symptoms in male patients with androgenetic alopecia at both the 4- and 6-month milestones, with no negative effects when compared to using Minoxidil alone [130]. The effect of botulinum toxin A on the regeneration of hair follicle cells was also explored under continuous stress conditions in a pilot animal study [131]. This study evidenced reduced hair growth under stress conditions as well as in the BoNT treatment groups under stress compared to the control group. After 3 weeks, the telogen stage was seen under stress conditions, whereas the anagen stage was evidenced in the BoNT and control groups. Substance P (SP) immunoreactivity cell levels were increased under stress conditions compared to the groups receiving BoNT under stress conditions and to the control group. When stress was introduced, a delay in the hair follicle cell regeneration cycle was observed. Conversely, when BoNT was injected subcutaneously, the regeneration cycle returned to nearly normal, highlighting the beneficial role of BoNT in treating hair loss.

### 8.2. Cardiac Effects of BoNTs

#### 8.2.1. Beneficial Effects in Experimental Models of Atrial Fibrillation

BoNTs are responsible for a temporary suppression of cholinergic transmission, leading to inhibition of atrial fibrillation (AF). An in vivo study confirmed the suppression of atrial ganglionic plexi by BoNT, inducing autonomic electrical remodeling and providing long-term suppression of AF, indicating the benefit of BoNTs and the critical role of GPs in AF [132]. Comparable findings were observed in a sheep in vivo model, where BoNT injections decreased the susceptibility of atrial tissue to AF and diminished the vagal impact on atrial ERP shortening when compared to baseline levels. Additionally, direct BoNT injections did not lead to any myocardial dysfunction or adverse histological effects [133]. Short- to mid-term effects of BoNT on AF were explored in 23 mongrel dogs where sinus node and atrioventricular node epicardial fat pads were injected with BoNT/A (Botox, 50 U per fat pad). Vagal stimulation was reduced, and the atrial effective refractory period was affected one week after the BoNT injection but disappeared at 2 and 3 weeks, representing a novel therapeutic option for clinical conditions such as post-cardiac surgery AF [134]. In addition to beneficial effects on atrial fibrillation, BoNT/A can reduce bradycardia due to parasympathetic ganglionic activation. The effects of BoNT/A injections into the sinoatrial (SA) fat pad were investigated in anesthetized dogs to assess impact on bradycardia following stimulation of the preganglionic parasympathetic nerves. Stimulating the vagal nerves in succession accelerated the inhibitory effect of botulinum toxin. This suggests that injecting BoNT/A into the sinoatrial fat pad inhibits bradycardia caused by parasympathetic ganglionic activation in a dog’s heart [135].

#### 8.2.2. Effects of BoNT/A in Postoperative Atrial Fibrillation

Postoperative atrial fibrillation is a critical complication of cardiac surgery, associated with prolonged hospitalization, increased risk of stroke, and mortality. Botulinum toxin type A injection within the autonomic ganglia can reduce arrhythmogenic activity, representing a promising strategy to prevent postoperative atrial fibrillation [136,137]. Nevertheless, there was an indication that administration of high doses of BoNT/A can increase the risk of systemic diffusion and adverse effects, but no consensus was formulated regarding the maximum tolerated dose. The use of BoNT/A in adult patients at doses up to 12 units/kg did not induce significant variations in heart rate variability, showing that BoNT/A represents a safe therapy in spastic hemiplegic stroke survivors [138].

Animal models have established the moderating effects of botulinum toxin on atrial fibrillation. Randomized double-blind studies were performed in patients presenting with atrial fibrillation to assess the efficacy and safety of botulinum toxin injection. Patients were followed for 1 year using an implantable loop recorder. The occurrence of postoperative atrial fibrillation within 30 days following coronary artery bypass grafting was notably decreased in patients treated with a BoNT injection. Administering botulinum toxin into the epicardial fat pads during the surgery significantly alleviated atrial tachyarrhythmia in both the immediate period and throughout a 1-year follow-up, with no serious adverse effects reported [139,140]. The treatment of patients undergoing cardiac bypass with BoNT injections into the epicardial fat pads resulted in a sustained and substantial reduction in atrial tachyarrhythmia incidence during 3-year follow-up and a significant reduction in hospitalizations [141].

The prevalence of overactive bladder (OAB) is increasing with age and can be associated with cardiac arrhythmia, but anticholinergic drugs clinically used to treat OAB can unfortunately alter the cardiovascular system, causing tachycardia. The influence of intradetrusor BoNT/A injections on electrocardiogram (ECG) parameters was investigated in patients with overactive bladder, showing that intravesical BoNT/A injections did not significantly influence the ECG. Thus, BoNT therapy was shown to be safe in patients with overactive bladder [142]. 

## 9. Effects of BoNTs on Parkinson’s Disease

A study examining the central effects of botulinum neurotoxin A (BoNT/A) in a rat model of Parkinson’s disease has demonstrated an enhancement in the motor behavior of the rats following cerebral administration of BoNT/A; the treatment targeting the striatum with BoNT/A reduces motor impairments and alters the density of D1 and D2 receptor-binding sites in the Parkinson’s disease rat model [143]. It was also hypothesized that BoNT/A-application into the striatum of hemi-Parkinsonian rats would block the excessive cholinergic transmission, leading to reduced pathological effects. Previous studies have shown that when BoNT/A is applied intrastriatally, it completely eliminates apomorphine-induced rotations for up to 6 months [144,145]. This application of BoNT/A functions not only as an inhibitor of ACh release but also influences other neurotransmitter receptors, particularly affecting D2 receptor expression and the circuitry of the basal ganglia [146,147].

The structural effect of unilateral intrastriatal BoNT/A injection was assessed in the naïve mouse brain to extend the potential applications of BoNT/A treatment to Parkinsonian mouse strains. The intrastriatal injection of BoNT/A did not modify the number of immunoreactive choline acetyltransferase interneurons but did modify the numeric density of the choline acetyltransferase-immunoreactive neurons. Botulinum-induced varicosities (BiVs) at a dose of 25 pg increased from 1 to 3 months after BoNT/A, followed by a time-dependent decrease. In contrast to rats, no tyrosine hydroxylase-ir BiVs were found in mouse striata receiving BoNT/A [148]. Injections of BoNT/A2 in mice resulted in complete relief from grand mal seizures for 4 days in half of the amygdala-kindled mice. Additionally, there was a noticeable reduction in the severity of induced seizures for at least 18 days following the injections. This suggests that BoNT/A2 could be a promising therapy for epilepsy [149].

## 10. Discussion

This review concentrated mainly on the different actions of BoNT/A, since data on other serotypes is notably under-represented due to the scarce availability of source data. Nonetheless, to ensure thoroughness, the review included available data, such as the impact of BoNT/B on intestinal peristalsis, the reduction in neuropathic pain, and its effects on vascular ischemia-reperfusion injury. The focus of the research presented has predominantly been on BoNT/A due to scarcity of data on other serotypes.

The main limitation of this study is that the cleavage of SNAP-25 by BoNT/A can independently alter Ca^2+^ levels, beyond just causing a blockade in neurotransmitter release. While many actions of BoNTs have been identified, further research is necessary to confirm that these new effects are not linked to changes in Ca^2+^ levels. Since SNAP-25 cleavage impacts the interaction between the SNARE complex and synaptotagmin, some effects previously attributed to non-SNAP-25 mechanisms might actually be influenced by Ca^2+^ levels and changes in SNARE interaction with ion channels.

Indeed, throughout this review, it is difficult to differentiate which toxin’s actions are in fact likely related to SNAP-25 cleavage, and which actions are probably not related. For example, the bladder indications of BoNT/A are probably related to the classical SNARE-mediated toxin action, although a significant decrease in noradrenergic neurons and an increase in neurons expressing nNOS were observed.

The influence on gene expression is considered unlikely to be connected to SNAP-25 cleavage mechanisms; however, additional research is also necessary to confirm that SNAP-25 cleavage does not impact gene expression. Previous studies have already established the effects on Rho GTPases independently of SNAP-25 cleavage, indicating that BoNT/A also targets the RhoB pathway. Similarly, the effects of BoNT/A on cancer cells are unlikely to be related to SNAP-25 blockade since they are related to apoptosis and gene regulation. Regarding inflammation and secretions, it was previously established that PLA(2) and AA are indeed involved in neuro-exocytosis independently of SNAP-25 cleavage [43]. Nevertheless, there remain numerous unanswered questions about the antinociceptive mechanisms and other effects of BoNT/A that require further investigation today [68]. BoNT/A decreases glutamate release from cultured astrocytes, despite the absence of SNAP-25 in these cells. It also interferes with the activation of store-operated calcium currents in oocytes and HEK293 cells, which similarly do not express SNAP-25 [81]. The data published in the PLOS ONE Article and anti-cancer studies suggest that cellular responses to BoNT/A involve processes beyond immediate SNARE protein cleavage, driven in part by binding or entry mechanisms shared by the inactive holotoxin derivative [26].

Another limitation of the study involves the difference between the doses used in cell culture and those used in vivo. The effects of toxins observed in cell culture often do not match the in vivo effects, as the doses used in vivo are lower due to exquisite tissue sensitivity. This discrepancy can be partially attributed to the inactivity of neurons in vitro, whereas neurons in a living organism are continuously stimulated and embedded in a full neuronal network.

This review does not aim to exaggerate the significance of effects that are not mediated by SNAP-25 in vivo. It is important to interpret the results carefully, as the influence of SNAP-25 cleavage has only been examined in a limited number of studies. The various SNAP-25 non-dependent pathways covered in the review are summarized in Table 4 below.

## 11. Conclusions

Nowadays, a wide range of applications of BoNT/A have been tirelessly explored in clinical practice. However, a wealth of mechanisms underpinning the multiple relationships between muscle hyperactivity, neuronal sprouting, endothelial cell functions, inflammation, neuropathic pain, Parkinson’s disease, and migraine treatment need further exploration.

Since the first clinical applications of BoNTs envisioned by Dr Justinus Kerner, who proposed the use of this “sausage toxin” to treat hypersecretion disorders, a long journey has been undertaken to discover diverse clinical applications. Beyond the SNAP-25 cleavage, BoNT influences gene expression, restores the neuroimmune balance, targets the Rho pathway, inhibits the growth of cancer cells, reduces secretions and inflammation, reduces hypertrophic scars, modulates collagen production, inhibits the release of nociceptive peptides, reduces neuropathic pain, supports neuronal regeneration, attenuates sympathetic vasoconstriction, attenuates atrial fibrillation, and improves motor behavior in Parkinson’s disease.

This review brings fresh insights into the diversity of the mechanisms of action of BoNT/A, opening new avenues of research into therapeutic options for patients. With the varied influence on gene expression, cancer models, inflammation, skin regeneration, neuropathy, vascular relaxation, and atrial fibrillation, new promising research avenues are emerging in the field of BoNTs for therapeutic applications in cancerology, inflammation, skin disorders, neuropathies, and cardiovascular diseases. The physical healing of musicians treated with BoNT constitutes a prefiguration of this myriad of actions of BoNT/A. However, significant gaps in knowledge remain regarding the mechanisms of action and, more specifically, how to address the role of SNAP-25 cleavage in the variety of actions.

## Figures and Tables

**Figure 1:**
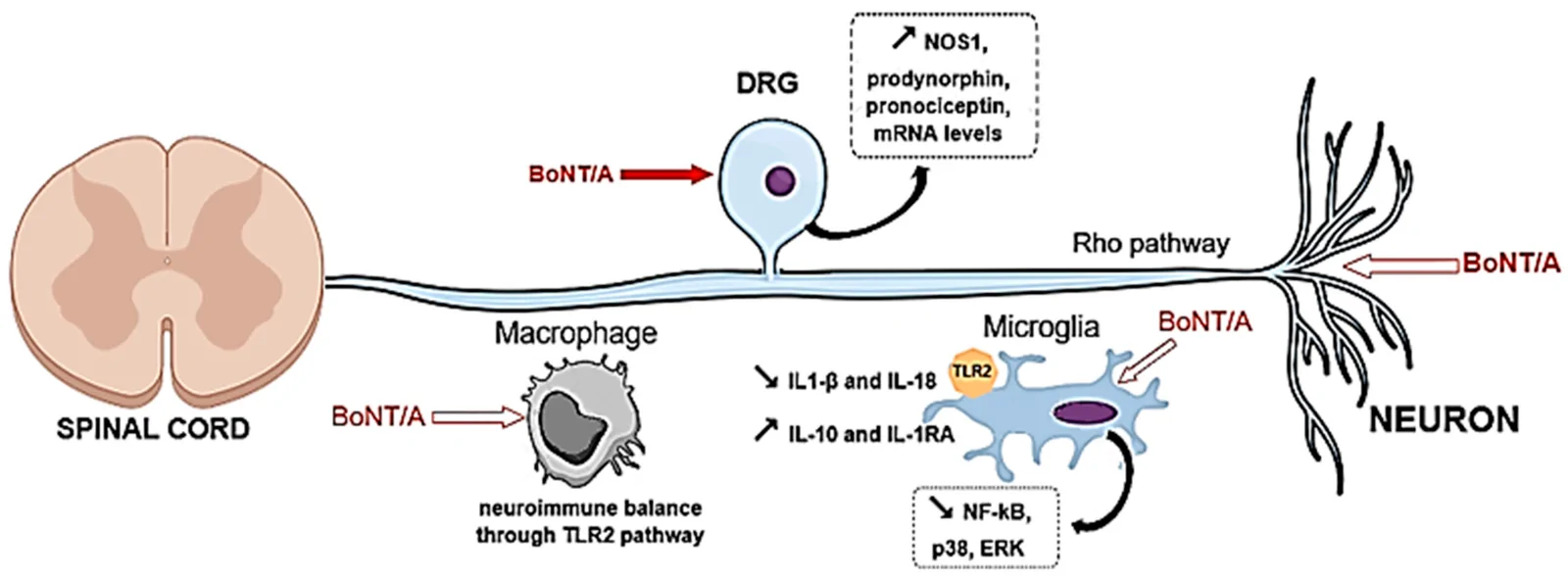
Modulation of neuroinflammation by BoNT/A on dorsal root ganglia (DRG) and microglia.

**Figure 2:**
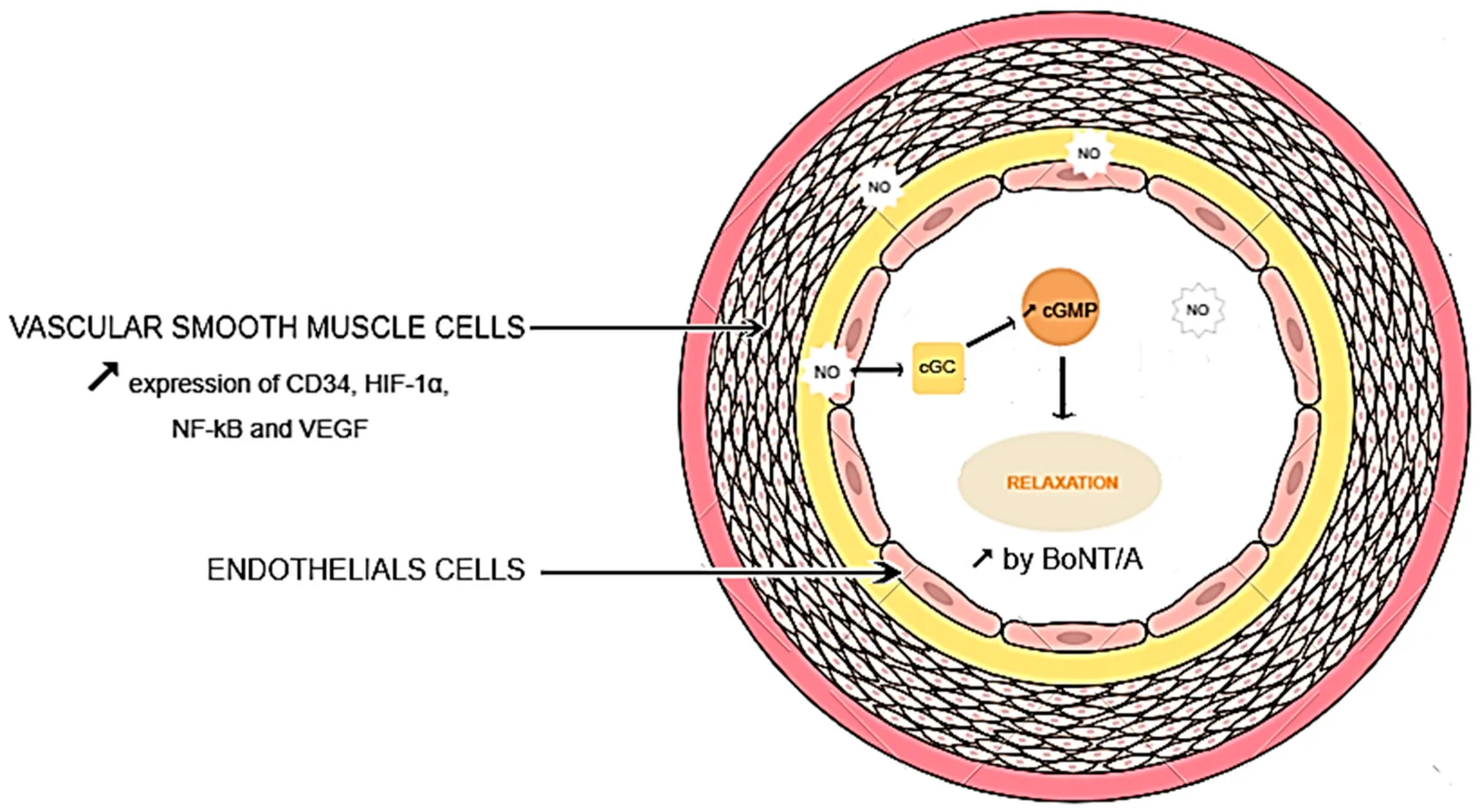
BoNT/A suppresses arterial vasoconstriction by up-regulation of the eNOS/sGC/cGMP pathway.

**Table 1:** Modulation of genes in different cell types.

Mechanism	Target	References
Adjustment of genes associated with neuroinflammation, the ubiquitin-proteasome degradation system, phosphatidylinositol, calcium signaling, as well as those related to focal adhesion, cell adhesion molecules, and pathways involving adherens and gap junctions.	SH-SY5Y cells	[27]
Influence on NOS1, prodynorphin, and pronociceptin mRNA levels in the DRG	Dorsal root ganglions	[22]
Restoration of neuroimmune balance through TLR2 pathway	Macrophages	[23,24]
Inhibition of the expression of pro-inflammatory factors by modulation of NF-κB, p38, and ERK1/2, and increased expression of TLR2 and its adaptor protein, MyD88	Microglia	[25]
Changes in 492 genes for BoNT/A and 682 genes for the atoxic derivative, after 2 weeks of exposure	Human iPSC-derived neurons	[26]
BoNT inhibits neuroexocytosis by also targeting the RhoB pathway	Nerve growth factor-treated PC12 cells	[28]
Inhibition of the growth of LNCaP human prostate cancer cells in vitro and in vivo, inducing apoptosis	Human prostate cancer cells	[30,31]
Up-regulation of 167 genes and downregulation of 60 genes involved in focal adhesion, cell adhesion molecules, and gap junction pathways	Carcinoma cell lines	[27,32,33].
Cytotoxic activity in T47D cells compared with MCF10A normal cells by eliciting caspase-3 and -7 dependent apoptotic processes	Breast cancer cell lines T47D	[34]
Profound decrease in the number of noradrenergic neurons expressing NPY, VIP, SOM, and GAL, and increase in the number of neurons expressing nNOS and L-ENK	In vivo (Pigs)	[37]

**Table 2:** Effects on skin regeneration.

Mecanism	Tissue	References
Inhibition of growth factor expression and decrease in connective tissue growth factor	Hypertrophic scars	[49]
Modulation of cell cycle	Hypertrophic scars	[50]
Decrease in TGF-b1 protein	Fibroblasts	[52]
Inhibits the growth of fibroblasts derived from SC and reduces the expression of α-SMA and myosin II	Fibroblasts derived from scar contracture	[53]
Decreased expression of collagen III, increased expression of MMP-1, and heightened enzymatic activity of MMP-2	Keloids	[56]
Blocking of TGF-β1 signaling	Capsule contracture	[59]
Increased expression levels of CD31 and vascular endothelial growth factor (VEGF)	Adipose tissue graft	[62]
Inhibition of mast cells degranulation	Skin rosacea	[65]

**Table 3:** Differences in the expression of biological markers associated with peripheral nerve injury in CCI-A15 and CCI-B7.5 vs. CCI-saline mice (Luvisetto 2022 [90]).

Target	Marker	CCI-A15	CCI-B7.5
Non-myelinating SCs	GFAP	↑	↓
Myelinating SCs	S100b	=	=
Peripheral myelin	P0	↓	↓↓
Peripheral myelin	PMP2	↓↓↓	↓↓
Mast cells	CC1	↑↑↑	↑
Macrophages	CD11b	↑↑↑	=

CCI: Chronic constriction injury; A15: 15 pg/paw; B7.5: 7.5/paw; GFAP: Glial fibrillary acidic protein; S100b: Calcium-binding protein B; PMP2: Peripheral Myelin Protein 2; CD11b: integrin alpha M; ↑: increase; ↓: decrease; =: no effect.

**Table 4:** Integrated SNAP-25 non-dependent pathways.

Pathways	Mecanims	References
Effects on gene expression	-Inhibition of the expression of pro-inflammatory factors by modulation of NF-κB, p38, and ERK1/2	[25]
	-Increased expression of TLR2 and MyD88	[25]
	-Depression in the number of the noradrenergic neurons expressing NPY, VIP, SOM and GAL and increase in the number of the neurons expressing nNOS and L-ENK	[37]
Secretions and inflammation	Block of the release of AA in a PC12 cell line model	[41,42]
Intestinal peristalsis and microbiota composition	Alterations in the release of mucin leading to a disruption of the protective barrier against intestinal infections	[45]
Dermal fibroblasts and regeneration	-Inhibition of the growth of hypertrophic scar-derived fibroblasts and decrease in connective tissue growth factor (CTGF)	[49]
	-Down-regulation of collagen III expression, up-regulation of MMP-1, and increased MMP-2 enzymatic activity	[56]
Reduction in skin inflammation	Inhibition of mast cells degranulation	[65]
Effects on pain and neuropathy	-Deletion of SP/ NKA and NK1R genes in acute and chronic inflammatory pain as well as neuropathic pain	[76]
	-Down-regulation of TRPV1 and TRPA1 -Inhibition of nociceptive peptides released at the injection site and the suppression of expression of inflammatory cytokines	[80,83]
	-Reduction in P2X7 receptor density	[87]
Neurogenic actions	-Higher expression of ENK, GFAP, S100β, GAP43, NF200, BDNF, and NGF	[105]
Neurogenic bladder	-Decreased levels of sensory receptors P2X3 and/or TRPV1	[113]
	-Profound decrease in the number of noradrenergic neurons expressing NPY (neuropeptide Y), VIP (vasoactive intestinal polypeptide), SOM (somatostatin), and GAL (galanin), and an increase in the number of neurons expressing nNOS (neuronal nitric oxide synthase) and L-ENK	[37]
Vascular benefits	-Higher expressions of CD34, hypoxia-inducible factor 1 alpha (HIF-1α), nuclear factor-kappaB (NF-kB), and vascular endothelial growth factor (VEGF)	[127]
	-Regulating smooth muscle calcium sensitization via the eNOS/sGC/cGMP pathway	[129]
Cardiac effects	-Moderating atrial fibrillation	[132,133,134,135,136,137,138,139,140,141,142]

## Data Availability

No new data were created or analyzed in this study. Data sharing is not applicable to this article.
